# A Probable Solution for Femininism

**Published:** 1914-03

**Authors:** Alfred Russell Wallace


### A Probable Solution for Femininism.

There has been no advance either in intellect or morals, from the days of the earliest Egyptians to the keel-laying of the dreadnought.

Now it is in this inherent and unchangeable character itself that tends to be transmitted to offspring, and this being the case, there can be no progressive improvement in character without some selective agency tending to such improvements.

The higher intellectual or moral powers are so rarely of life preserving value, and are not infrequently the reverse, that they are not cumulative, though they are hereditary. For the evolution of man's moral and intellectual nature, then, we must look to some selective agency. Such an agency will become operative when women achieve real freedom of choice in marriage. Then we shall find a system of truly natural selection will come spontaneously into action, which will steadily tend to eliminate the lower, the less developed, or in any way defective types of men, and will thus continuously raise the physical, moral and intellectual standard of the race.—Dr. Alfred Russell Wallace.

